# What Defines an Effective Anti-Tobacco TV Advertisement? A Pilot Study among Greek Adolescents

**DOI:** 10.3390/ijerph7010078

**Published:** 2010-01-08

**Authors:** Constantine I. Vardavas, Emmanouil K. Symvoulakis, Gregory N. Connolly, Evridiki Patelarou, Christos Lionis

**Affiliations:** 1 Clinic of Social and Family Medicine, Department of Social Medicine, Faculty of Medicine, University of Crete, Heraklion, 71003, Greece; E-Mails: symvouman@yahoo.com (E.K.S.); lionis@galinos.med.uoc.gr (C.L.); 2 Harvard School of Public Health, Harvard University, Boston, MA 02115, USA; E-Mail: gconnoll@hsph.harvard.edu; 3 University Hospital of Heraklion, Crete, 71003, Greece; E-Mail: patelarou@edu.med.uoc.gr

**Keywords:** smoking, prevention, mass media, health education, adolescents, tobacco control, anti-tobacco, counter advertising

## Abstract

As the Framework Convention on Tobacco Control (FCTC) calls for public health awareness on tobacco use, mass media campaigns should be appropriately designed so as to maximize their effectiveness. In this methodological pilot study, 95 Greek adolescents (mean age 15 ± 1.8 years), were shown seven different anti tobacco ads, and asked to rate the ad theme, message and emotional context on a 1−7 Likert scale. Health related ads were rated the highest, and as identified through the logistic regression analysis, adolescents who perceived an ad to be emotional or to have a clear message that was relevant to them, were more likely to rate the ad as more effective. The strong agreement between the above findings and the existing literature indicates the applicability of this pilot study’s methodological approach.

## 1. Introduction

Mass media have played a key role in the historical increases and decreases in tobacco use globally, and remain a powerful tool for public health advocates, as an avenue of projecting health related messages [1]. Through this aspect it has been suggested that to be influenced by a message, an audience must be exposed to it, pay attention to and understand it, and develop a cognitive or affective response [2]. Within the context of the theory of planned behavior (TPB) and the theory of reasoned action (TRA), an individual’s intention to act is a strong predictor of behavior, as long as the individual perceives that he or she has control over the behavior [3,4]. This intention to act can be influenced by mass media campaigns. Research conducted to date on the main characteristics that influence the effectiveness of a mass media campaign has indicated a plethora of possible influential factors. Even if many public health campaigns have focused on increasing the individuals knowledge about the risk of disease development, it is the individual’s feelings (about activities with health consequences) that predict behavior better than the knowledge of the risks incurred [5].

These above points must be taken into account when designing effective antismoking campaigns, and even more so when preventing adolescent smoking is the key objective. Among adolescents, the effectiveness of a mass media campaign has been found to depend on a number of factors including emotional content, theme and message, with previous research indicating that themes that depict short term health effects and social norms to be more influential than those that depict long term health effects [6]. Furthermore, the emotional impact of the projected message has also been stated to play a key role in a mass media campaign’s perceived effectiveness [7].

Greece has recently initiated a stricter tobacco control plan that will aim to regulate tobacco advertising, smoke free areas and in parallel help changing smoking norms and the acceptance of smoking among the local population of Greece [8]. However, despite this new challenge, there is currently a lack of regional translational research on how to promote the population’s adherence to the changes of the upcoming tobacco control policy. Taking this fact into account, with this pilot study we aimed to investigate into the different factors that mediate the effectiveness of an anti-tobacco advertisement and how these factors can be successfully measured by applying a simple methodological approach among a convenience sample of Greek adolescents.

## 2. Methodology

### 2.1. Sampling Methodology—Ethical Considerations

This pilot study was conducted in Heraklion, Crete, Greece between the months of April to June 2008, after having obtained approval by the Ethics committee of the University Hospital of Crete (Protocol number: 1252-12/2/2008) and with the support of the Health Promotion Office, of the Department of Secondary Education of Heraklion, Greece. A convenience sample of 95 adolescents participated in the study, aged 12−19 (Mean age 15.1 ± 1.8, of which 70% were female, due to the higher percentage of girls in one of the two schools) currently enrolled in five classes in two high schools in Crete, Greece. The school teachers were briefed and gave verbal consent while written consent was obtained by both the participating adolescents and their parents after having received a written invitation and an explanatory letter. Following the survey, all adolescents participated in an interactive class workshop through which the projected ads were discussed.

### 2.2. Questionnaires, Theory, and Procedures

In this analysis, the participants were asked to complete two questionnaires. Initially they were requested to fill in a shortened version of the Global Youth Tobacco Survey (GYTS) questionnaire, which has been translated into Greek and applied successfully in the context of a previous study [9]. This shortened version of the original GYTS questionnaire collected information on demographical characteristics, smoking experimentation, current smoking habits, tendency to initiate or quit, as also information on their perceived exposure to pro and anti-tobacco advertisements over the past month. According to this questionnaire, the participating adolescents were categorized as smokers (or experimenting smokers) if they reported any tobacco use during the previous month. In the analysis we categorized those who had experimented with cigarettes in the past month together with those adolescents who report smoking more frequently. This was performed so as to clearly distinguish them from those who were not currently experimenting with tobacco as also to increase the size of the comparable study groups in the analysis. Furthermore, respondents were defined as non susceptible to smoking if they answered “definitely not” to the question “At any time during the 12 months, do you think that you will smoke a cigarette?”. Responses to this question were rated on a four point scale (1 = no, definitely not”, 2 = maybe not, 3 = maybe yes, 4 = yes, definitely) and those coded 2−4 were regarded as susceptible non smokers. Although this coding does not mean that the adolescents will smoke, they do not rule out the possibility that they might smoke in the next year, which is an indicator of their attitude towards smoking and their perceived behavioral control of their ability not to smoke. Moreover, smoking resistance self-efficacy is a consistently significant predictor of adolescent smoking [10].

During the next section of the survey the adolescents were shown in random order the seven different anti-tobacco TV advertisements. The viewing took place in a specifically prepared video room, supported by the necessary audio-visual projection equipment, in which 10 adolescents would enter at a time, without allowing for those who had viewed the ads to communicate to those who had not. The 7 different anti-tobacco advertisements that were shown in random order to the participating adolescents are described in Table 1 and represent different types of anti-tobacco advertisements, that cover a broad range of topics and which differ significantly in context, theme, origin and emotional tone. Each anti-tobacco ad was shown twice, after which the adolescents were requested to fill in a slightly modified version of the “Global dialogue for effective smoking campaigns” questionnaire that has been effectively used to qualitatively evaluate the perceived effectiveness and emotional context of anti-tobacco advertisements, and if freely available from the internet in a number of languages (Global dialogue for effective stop smoking campaigns: Campaign development tool kit: Appendix 3.1) [11].

Through this questionnaire, the participants were asked to respond to selected questions in regards to the characteristics of the projected anti-tobacco TV advertisements. Initially they were asked if the projected TV-advertisement was “clear to them”, “that had a message important to them”, “that said things that were hard to believe”, if it made them “stop and think”, “curious” and “if it told them something new”. These questions aimed to evaluate their response to the projected themes and topics and how it influenced their attitude towards smoking through the theory of planned behavior (attitude-subjective norms). All responses to the above were provided on 1−7 Likert type scale (1 = strongly disagree to 7 = strongly agree). Furthermore, for each advertisement they were requested to provide their perceived emotional status and respond how much each video made them feel sad, angry, happy and scared (4 separate responses), and how they regarded it as funny, boring, emotional and powerful (another 4 separate responses). Again all of the above responses were provided on the same 1−7 Likert type scale. During data recording, all responses to the Likert scale questions provided by the adolescents were subsequently reduced to the dichotomous level, by combining the 1−4 responses as “neutral or negative” and the 5−7 responses as “positive”.

### 2.3. Statistical Analysis

Initially univariate analyses such as chi-squared tests and student t-tests were performed between the descriptive characteristics of the study population and their basic responses. All *p*-values are based on two-sided tests and a significance level lower than 5% was defined. Likert scale variables were regarded as ordinal and thus no means or standard deviations were extracted. Logistic regression analyses allowed for the investigation into how the adolescents perceived the ads as powerful according to the ads characteristics and emotional context. Greater odds ratios (OR) imply higher likelihood rates of the advertisements effectiveness. The above results were adjusted for the participants age (<15 years *vs.* >15 years), current smoking status and gender. Where applicable, 95% confidence intervals are also provided. The statistical analysis was performed with the statistical packages SPSS 16.0. and STATA 10.0.

## 3. Results

In total, 95 adolescents participated in this pilot study, out of which 67 were female (70%) and 28 male (30%). The mean age of the participants was 15.1 (SD: ± 1.8) years of age (range 12−19), with boys slightly younger than girls (14.4 *vs.* 15.4, *p* = 0.02). Forty six percent (44/95) reported having ever tried a cigarette, while 33.3% (32/95) reported current tobacco use (more than one cigarette during the last month). Furthermore, 30% responded to have been offered a cigarette by an associate of the tobacco industry during a tobacco industry promotional activity. Regarding smoking susceptibility, among the non smokers, the majority were self reported as non susceptible to smoke within the next 12 months, while 14.1% (n = 9) of the non smokers indicated an intent to experiment with tobacco during the next 12 months. The frequency and number of adolescents that reported that they “agree to strongly agree” to each posed question, was found to depend on both the meaning of the question and the anti-tobacco ad that they referred to, as illustrated in Table 2 below.

As depicted in Table 3, we investigated into how the adolescents perceived the viewed mass media campaigns as effective by smoking status, through rating them with a 5−7 score (effective- very effective) on the Likert scale in response to the question, “How effective is this ad as part of an anti-tobacco media campaign”.

As seen above, the analyses revealed that the most effective ad shown was “PAM” (96.8% rated it with 5−7), followed by “TAR” and “ARTERY” (89.6% and 84.2% of the adolescents rated them with a 5−7). Smoking status was not found to alter significantly the adolescents’ response, with the exception of “HELP-secondhand smoke”, to which non smoking adolescents were found to rate it higher in comparison to their peers that smoke (77.8% *vs.* 43.8%, respectively, *p* < 0.001).

The results of the logistic regression analysis performed to explore the relationship between regarding an ad as effective and certain ad characteristics, are depicted in Table 4 below. Controlling for the adolescents age, gender and current smoking status, the ads clarity was noted as an important characteristic, as adolescents that rated an ad as clear (with a 5−7 on the Likert scale), were also more likely to rate it as more effective (odds ratio, OR 7.3; 6.3; and 6.2 for the ads “HELP-PEERS”, “BAR SMOKING” and “ARTERY” respectively). Additionally adolescents who rated the ads highly in response to the question “Does the ad have a message that is important to me?” were also more likely to regard the ad as more effective, with an OR of 12.4 for “ARTERY” and 11.7 for “HELP-secondhand smoke”. A mass media anti tobacco campaign was also rated as more effective by the adolescents, if it made them “stop and think”, with statistically significant OR’s identified for almost all projected anti tobacco advertisements.

The results of the second logistic regression that was performed so as to explore the role of the perceived emotional context on the ads perceived effectiveness are depicted in Table 5.

As seen above, after controlling for the respondents age, gender and current smoking status, the emotional context of a mass media campaign was found to play an important role into how the adolescents perceive it to be effective. In general adolescents who rated the anti tobacco advertisements as more emotional were more likely to rate them as more effective. Those who felt sad after watching the “TAR” advertisement were almost 10 times more likely to rate the ad as effective (OR: 9.9), while similar findings were also noted for “ARTERY” (OR: 6.8), “BAR SMOKING” (OR: 6.5) and both HELP campaigns (OR: 12.7 and OR: 5.3 respectively). Feeling angry or scared, as a consequence of the projected anti-tobacco advertisement was also a strong factor that was found to influence the campaigns perceived effectiveness. Moreover on the other hand, rating the ad as happy, boring or funny was not found to be related to the ads effectiveness. In addition to the above, further details can be provided upon request, from the corresponding author (CIV).

## 4. Discussion and Conclusions

The results of this pilot study suggest that among youth, the effectiveness of a mass media anti-tobacco TV campaign is strongly related to the ads clarity, relevance to youth and its ability to make them “stop and think”. In the emotional domain, making an adolescent feel sad or angry was strongly related to the messages self rated effectiveness, while rating an anti-tobacco advertisement as happy, funny or boring was not. Comparing the international literature with our study’s findings we identified that the role of the emotional context of an anti-tobacco campaign appears to be beyond national differences with audiences acting similar despite the geographical and cultural diversity involved [12,13]. This strong agreement between this studies findings and the existing scientific literature indicate the applicability of the methodological approach used in this pilot study.

Social determinants and social and personally subjective norms, tobacco industry manipulation and the health consequences of active and passive smoking are the main themes that have been adopted over the past years in community based mass media campaigns and which differ significantly in message and character [14,15]. Extensive research on the ideal content of a mass media anti-tobacco campaign have indicated that emotionally intensive campaigns are more effective than unemotional advertising in achieving the same level of recall among youth [12], and are regarded as more persuasive and more effective in emphasizing the anti-tobacco message among adolescents, a finding also indicated through our pilot results among Greek adolescents [16]. Within the context of this study, the ads that were rated as more emotional and more effective were those that mainly depicted health consequences (“PAM”, “ARTERY” and “TAR”), a finding that has also been found among US adolescents. Indeed research by Shadel *et al*., has indicated that antismoking advertisements that emphasized the effects of smoking were related with the strongest smoking resistance self efficacy in adolescents in comparison to other themes [17]. Moreover, our findings indicated that anti-tobacco advertisements that portray social norms (*i.e.*, endangering others or the negative social circumstances surrounding smoking) were also perceived as effective among youth; such an example was the “HELP-SHS” advertisement [18].

Sadness, anger, fear and empathy are among the emotional domains that influence the likelihood that a message will be recalled and perceived as a stronger predictor of a campaign’s effectiveness [11,19]. In line with the above, adolescents who believed that the anti-tobacco advertisement was more emotional were up to seven times more likely to subsequently perceive it as effective. This finding is corroborated by the fact that adolescents who believed that the anti-tobacco campaign conveyed an important message to them, or made them “stop and think” were also more likely to rate the ad as more powerful in comparison to their peers who were rather indifferent to the conveyed message. Previous relative research among of our study group using Greek anti tobacco advertisements of multiple themes had also indicated that adolescents were more likely to perceive an anti tobacco advertisement as more effective if they found it to convey a clear important message of relevance to them [20].

As this is a pilot study, its main limitation is in the generalisability of the results due to the small convenience sample that was used; a fact that should be taken into account in subsequent studies at a regional level. Furthermore, it is possible that the novelty of the protocol itself could have influenced the adolescents to respond differently than if they were to view the anti-tobacco advertisements on television, a fact that we could not investigate into. We must state that the adolescents self reported perceptions of the anti-tobacco advertisements was a proxy of their current perception and thus we were not able to investigate into the real-life effectiveness of these media campaigns as pre and post surveys at a national level would be appropriate for such an investigation. Moreover we must state that we were not able to control for broadcast language as a confounder in the analysis, however in Greece the majority of television programs are in English and subtitled, similarly to the way the study’s advertisements were projected the majority of Greek adolescents.

This pilot study provides an insight into the methodological approach that can indicate the characteristics and traits that could make an anti tobacco advertisement effective, a fact that should be taken into account when designing youth oriented tobacco control campaigns. As the Framework Convention on Tobacco Control, states in article 12 that “*Each party shall promote and strengthen public awareness of tobacco control issues, using all available communication tool, as appropriate*”, it is essential that nations adopt comprehensive and effective mass media campaigns that are tailor fitted to each country’s needs [21]. Taking the above into account the applied methodological protocol that we adopted and further developed, can be used as a quick low cost research approach for gathering local data that can support the design of a mass media tobacco control program at a national level.

## Figures and Tables

**Table 1.** Origin, title and context of the anti-tobacco advertisements evaluated.

**Origin**	**Title**	**Language**	**Key Message**
European Union (2005)	Help – peer pressure to smoke	Greek	Peer pressure plays a key role in smoking experimentation.
European Union (2005)	Help – second hand smoke exposure	Greek	Children’s exposure to second hand smoke at home is annoying.
Australian NSW (1992)	Artery	English with Greek subtitles	Cigarette smoking blocks the aorta with fatty deposits.
Australian NSW (1992)	Tar	English with Greek subtitles	Cigarette smoking creates a build up of tar in the lungs.
United States MA (1999)	Camel	English with Greek subtitles	Camels do not smoke cigarettes. This is a spoof on Camel ads.
United States MA (1988)	Pam Laffin	English with Greek subtitles	Smoking causes emphysema.
Greece (2007)	Bar smoking	Greek	Secondhand smoke exposure inside bars is annoying and hazardous.

NSW: New South Wales; MA: Massachusetts.

**Table 2.** Percentage and number of adolescents that rated each question with a 5−7 score on.

	**HELP Peers**	**HELP SHS**	**Camel**	**Tar**	**Artery**	**Pam**	**Bar smoking**
	% (n)	% (n)	% (n)	% (n)	% (n)	% (n)	% (n)
**This ad…**							
Was clear	63.5 (61)	66.7 (64)	49.0 (47)	89.6 (86)	86.5 (83)	91.7 (88)	58.3 (56)
Had a message that is important to me	53.1 (51)	62.5 (60)	35.4 (34)	90.5 (86)	83.3 (80)	93.8 (90)	47.9 (46)
Said things that were hard to believe	16.7 (16)	16.7 (16)	15.8 (15)	49.0 (47)	47.9 (46)	49.0 (47)	14.6 (14)
Made me stop and think	43.6 (41)	58.5 (55)	38.3 (36)	77.1 (74)	72.9 (70)	78.1 (75)	30.2 (29)
Made me curious to know if it is true	18.1 (17)	25.8 (24)	23.9 (22)	52.1 (50)	46.3 (44)	51.6 (49)	19.1 (18)
Told me something new	12.5 (12)	22.9 (22)	12.6 (12)	55.2 (53)	56.3 (54)	66.7 (64)	13.7 (13)
Talked down to me	12.5 (12)	12.5 (12)	9.6 (9)	25.0(24)	19.8 (19)	19.8 (19)	11.8 (11)

**This ad made me feel….**							
Sad	20.0 (19)	40.9 (36)	8.0 (7)	52.2 (48)	51.6 (47)	80.9 (76)	15.2 (14)
Angry	16.3 (15)	21.3 (19)	14.9 (13)	37.8 (34)	36.3 (33)	44.4 (40)	21.3 (20)
Happy	10.9 (10)	6.7 (6)	23.9 (21)	7.8 (7)	7.7(7)	7.8 (7)	16.5 (15)
Scared	18.7 (17)	24.7 (22)	7.2 (6)	59.1 (55)	56.8 (54)	70.3 (64)	16.7 (15)

**This ad was….**							
Funny	8.7 (8)	9.2 (8)	61.1 (55)	8.8 (8)	7.8 (7)	4.3 (4)	28.3 (26)
Boring	14.3 (13)	14.8 (13)	16.9 (15)	9.0 (8)	6.7 (6)	11.0 (10)	21.3 (19)
Emotional	30.8 (28)	55.7 (49)	21.6 (19)	46.7 (43)	40.2 (37)	83.9 (78)	22.5 (20)

n: sample size.

**Table 3.** Percentage that rated the media campaigns as effective (5−7 score) by smoking status.

	**Total study population % (n)**	**Smokers % (n)**	**Non Smokers % (n)**	**p-value**
**Help peer pressure**	60.0 (57)	56.3 (18)	61.9 (39)	0.660
**Help secondhand smoke**	66.3 (63)	43.8 (14)	77.8 (49)	**0.001**
**Camel**	50.5 (48)	43.8 (14)	54.0 (34)	0.390
**Tar**	89.6 (86)	93.8 (30)	87.5 (56)	0.488
**Artery**	84.2 (80)	87.5 (28)	82.5 (52)	0.767
**Pam**	96.8 (92)	100.0 (32)	95.2 (60)	0.548
**Bar smoking**	55.4 (51)	63.3 (19)	51.6 (32)	0.372

n: sample size.

**Table 4.** Perceived effectiveness of a mass media campaign in relation to the ads characteristics as investigated through a logistic regression analysis (controlled for age, gender and smoking status).

	**HELP Peers**	**HELP SHS**	**Camel**	**Tar**	**Artery**	**Pam**	**Bar smoking**
	OR(95%CI)	OR(95%CI)	OR(95%CI)	OR(95%CI)	OR(95%CI)	OR(95%CI)	OR(95%CI)
	
**This ad…**							
Was clear	**7.3(1.9−28.3)**	**2.9 (1.0−7.8)**	**4.6(1.4−14.5)**	**4.8 (1.1−19.8)**	**6.2(1.6−23.9)**	1.7 (0.3−9.6)	**6.3 (1.6−24.2)**
Had a message that is important to me	**4.7 (1.5−14.7)**	**11.7(3.2–41.9)**	**4.1(1.4−11.6)**	**4.9 (1.1−22)**	**12.4(3.2−47.8)**	2.3 (0.4−13.9)	**7.8 (2.3−26.7)**
Said things that were hard to believe	1.3 (0.3−5.1)	1.7 (0.5−6.1)	3.1 (1.1−8.9)	2.9 (1.1−7.8)	1.8 (0.7−4.6)	1.0 (0.7−6.0)	0.6 (0.1−2.9)
Made me stop and think	**7.4 (2.0−26.5)**	**9.4 (3.0−29.2)**	**4.5 (1.5−12.8)**	**8.1 (2.7−24.3)**	**4.5 (1.6−12.3)**	**4.7 (1.6−14.3)**	n/a
Made me curious to know if it is true	1.2 (0.3−4.4)	1.7 (0.6−5.0)	2.0 (0.6−6.6)	**2.8 (1.0−7.2)**	1.4(0.5−3.5)	1.3(0.5−3.5)	1.4 (0.3−5.2)
Told me something new	0.6 (0.1−2.8)	1.5 (0.5−4.4)	1.4 (0.3−5.6)	**4.0 (1.5−10.7)**	**2.7 (1.0−7.1)**	1.3 (0.5−3.6)	3.4 (0.7−15.4)
Talked down to me	2.2 (0.5−8.9)	1.0 (0.3−4.6)	n/a	1.4 (0.5−4.2)	1.5 (0.4−4.9)	0.7 (0.2−2.3)	0.9 (0.1−4.9)

OR (95%CI): Odds Ratio and 95% Confidence Intervals, adjusted for age, gender and smoking status; n/a: not applicable due to small sample size.

**Table 5.** Perceived effectiveness of a mass media campaign in relation to the ads perceived emotional context as investigated through a logistic regression analysis (controlled for age, gender and smoking status).

	**HELP Peers**	**HELP SHS**	**Camel**	**Tar**	**Artery**	**Pam**	**Bar smoking**
	OR(95%CI)	OR(95%CI)	OR(95%CI)	OR(95%CI)	OR(95%CI)	OR(95%CI)	OR(95%CI)
	
**This ad made me feel….**							
Sad	**12.7(2.9−55.4)**	**5.3(1.9−14.9)**	1.4(0.2−9.9)	**9.9(3.1−31.0)**	**6.8(2.3−19.7)**	2.3(0.7−7.3)	**6.5(1.7−24.1)**
Angry	**4.9(1.3−18.1)**	**3.7(1.1−12.6)**	1.2(0.2−5.1)	**4.8(1.5−15.4)**	**6.1(1.7−21.0)**	**3.9(1.3−12.4)**	**6.9(1.9−24.4)**
Happy	0.2(0.0−2.2)	7.6(0.7−77.8)	1.7(0.6−5.3)	0.5 (0.1−2.5)	0.5(0.1−2.7)	2.7(0.3−24.7)	0.8(0.2−3.7)
Scared	3.2(0.9−12.1)	**3.8(1.1−12.8)**	n/a	**6.0(2.1−17.3)**	**5.9(2.1−16.2)**	**4.3(1.5−12.3)**	**5.8(1.5−22.0)**
**This ad was….**							
Funny	1.1 (0.1−8.5)	6.7(0.9−45.6)	1.0 (0.3−2.8)	0.4 (0.1−2.0)	0.6(0.1−3.5)	0.3(0.0−2.6)	1.0(0.3−3.6)
Boring	0.5(0.1−2.5)	0.1(0.0−0.8)	0.3 (0.1−1.9)	0.5(0.1−2.4)	2.8(0.3−26.8)	1.8(0.3−10.3)	0.3(0.0−1.5)
Emotional	**7.3(2.1−25.7)**	**4.4(1.5−12.7)**	0.4 (0.2−1.3)	**2.8 (1.1−7.6)**	**3.5(1.2−10.2)**	**3.7(1.1−12.6)**	**5.4(1.6−18.8)**

OR (95%CI): Odds Ratio and 95% Confidence Intervals, adjusted for age, gender and smoking status; n/a: not applicable due to small sample size.
